# Imputation to whole-genome sequence using multiple pig populations and its use in genome-wide association studies

**DOI:** 10.1186/s12711-019-0445-y

**Published:** 2019-01-24

**Authors:** Sanne van den Berg, Jérémie Vandenplas, Fred A. van Eeuwijk, Aniek C. Bouwman, Marcos S. Lopes, Roel F. Veerkamp

**Affiliations:** 1 Animal Breeding and Genomics, Wageningen University and Research, P.O. Box 338, 6700 AH Wageningen, The Netherlands; 2 Biometris, Wageningen University and Research, P.O. Box 16, 6700 AA Wageningen, The Netherlands; 3 Topigs Norsvin Research Center, 6640 AA Beuningen, The Netherlands; 4 Topigs Norsvin, Curitiba, 80420-190 Brazil

## Abstract

**Background:**

Use of whole-genome sequence data (WGS) is expected to improve identification of quantitative trait loci (QTL). However, this requires imputation to WGS, often with a limited number of sequenced animals for the target population. The objective of this study was to investigate imputation to WGS in two pig lines using a multi-line reference population and, subsequently, to investigate the effect of using these imputed WGS (iWGS) for GWAS.

**Methods:**

Phenotypes and genotypes were available on 12,184 Large White pigs (LW-line) and 4943 Dutch Landrace pigs (DL-line). Imputed 660 K and 80 K genotypes for the LW-line and DL-line, respectively, were imputed to iWGS using Beagle v.4.1. Since only 32 LW-line and 12 DL-line boars were sequenced, 142 animals from eight commercial lines were added. GWAS were performed for each line using the 80 K and 660 K SNPs, the genotype scores of iWGS SNPs that had an imputation accuracy (Beagle R^2^) higher than 0.6, and the dosage scores of all iWGS SNPs.

**Results:**

For the DL-line (LW-line), imputation of 80 K genotypes to iWGS resulted in an average Beagle R^2^ of 0.39 (0.49). After quality control, 2.5 × 10^6^ (3.5 × 10^6^) SNPs had a Beagle R^2^ higher than 0.6, resulting in an average Beagle R^2^ of 0.83 (0.93). Compared to the 80 K and 660 K genotypes, using iWGS led to the identification of 48.9 and 64.4% more QTL regions, for the DL-line and LW-line, respectively, and the most significant SNPs in the QTL regions explained a higher proportion of phenotypic variance. Using dosage instead of genotype scores improved the identification of QTL, because the model accounted for uncertainty of imputation, and all SNPs were used in the analysis.

**Conclusions:**

Imputation to WGS using the multi-line reference population resulted in relatively poor imputation, especially when imputing from 80 K (DL-line). In spite of the poor imputation accuracies, using iWGS instead of a lower density SNP chip increased the number of detected QTL and the estimated proportion of phenotypic variance explained by these QTL, especially when dosage scores were used instead of genotype scores. Thus, iWGS, even with poor imputation accuracy, can be used to identify possible interesting regions for fine mapping.

**Electronic supplementary material:**

The online version of this article (10.1186/s12711-019-0445-y) contains supplementary material, which is available to authorized users.

## Background

Use of whole-genome sequence (WGS) data is expected to improve the detection of quantitative trait loci (QTL) because such data are expected to contain most of the causal single nucleotide polymorphisms (SNPs), as was shown in dairy cattle populations by using WGS data of 234 bulls [1]. Improved QTL detection is even more important in pig breeding populations, since the QTL can be used to improve the accuracy of prediction in across- or multi-population scenarios [2], which is especially relevant for pig breeding programs where cross-breeding is practised.

To benefit from WGS data, a large population of animals with such data is needed. In spite of the decreasing costs of WGS [3], it is still relatively expensive to sequence a large number of animals. A less expensive approach to increasing the number of animals with WGS data is to impute from lower density SNP chips to WGS. With imputation, a smaller group of sequenced animals is required and the majority of the population can be genotyped with a lower density and cheaper SNP panel. Then, low-density SNP genotypes are imputed to WGS using the sequenced animals as reference population. In dairy cattle, several studies have shown that imputation to WGS is reliable even with a limited number of sequenced animals in the reference population, e.g. [4–7]. For example, for imputation from 777 K SNP genotypes to WGS, van Binsbergen et al. [4] obtained an imputation accuracy evaluated by cross-validation within the reference population of 0.83 with 90 sequenced Holstein bulls. Bouwman and Veerkamp [6] obtained an imputation accuracy, based on the correlation between true and imputed WGS (iWGS) of 0.83 for imputation using a multi-breed reference population consisting of 20 Holstein and 60 animals of three different breeds, and demonstrated that other breeds can improve imputation accuracy when the number of sequenced individuals from the target breeds is small. For most breeding companies, only a small number of animals is sequenced per line because, often, sequencing expenses must be divided across lines. In those cases, it might be beneficial to combine the WGS data across lines into one reference population for imputation. In addition to the size and composition of the reference population, accuracy of imputation depends on the size of the genotyping array, the extent of linkage disequilibrium (LD) in the reference and target population and the minor allele frequency (MAF) of the SNPs on the genotyping array [4–9]. As a result, combining populations for imputation may not provide sufficient imputation accuracy in all populations.

Inaccurate imputation can influence the results of follow-up analyses such as genome-wide association studies (GWAS), especially when the accuracy of imputation is ignored in those analyses. Two approaches to account for imputation errors are to filter SNPs based on imputation accuracy prior to analysis or to use dosage scores in the analyses. Using dosage scores means that all imputed SNPs are included in the analysis, although the power to detect associations with poorly imputed SNPs will be low compared to using accurately imputed SNPs. So far, there is little information on the accuracy of imputation to iWGS using data of a commercial pig breeding population and on the effect of using iWGS genotype or dosage scores in a GWAS. Therefore, the objectives of this study were (1) to investigate the accuracy of imputation to WGS in two pig lines using a multi-line reference population and a limited number of sequenced animals available for the target lines; and (2) to investigate the effect of using imputed WGS genotypes versus dosage scores in a GWAS on QTL detection.

## Methods

### Data

The dataset used in this study was provided by Topigs Norsvin. Phenotypes for the number of teats recorded after birth on 12,184 Large White (LW-line) and 4943 Dutch Landrace (DL-line) pigs were available. We investigated this trait because records were available for both sexes and its heritability is relatively high, i.e. 0.4 [10]. The phenotypic records were pre-corrected for fixed effects, i.e. herd-year-of-birth, sex, and the random effect of litter, which were estimated with a pedigree-based linear model by Lopes et al. [11]. After correction the average numbers of teats (± SD) were 15.68 (± 0.98) and 15.71 (± 1.04) for the LW-line and DL-line, respectively.

### Genotypes

For both the LW-line and the DL-line, Geneseek-Neogen GPPHD 80 K SNP genotypes were available for all animals with phenotypic records. In addition, genotypes from the Affymetrix Axiom porcine 660 K SNP chip were available for the 120 sires with the largest number of offspring in the set of genotyped animals of the LW-line. Using the 120 sires as reference population, within-line imputation from 80 K to 660 K SNP genotypes was performed for all phenotyped animals from the LW-line using FImpute v2.2 software [12] with the pedigree option. This resulted in an average imputation accuracy of ~ 0.99. Imputation from 80 K to 660 K SNP genotypes was not possible for the DL-line because none of the DL-line animals were genotyped with the 660 K SNP chip. To avoid confounding of results, imputation of the DL-line to 660 K using the LW-line 660 K reference population was not considered but for the subsequent GWAS, 660 K genotypes for the DL-line were generated from the iWGS dataset.

Quality control of the within-line genotypes (80 K and 660 K) consisted of excluding (1) insertions and deletions, (2) SNPs with a MAF lower than 0.01, (3) SNPs with a frequency lower than 0.1 for either one of the three genotypes, and (4) SNPs with missing map information (based on Sscrofa10.2).

### Reference population for whole-genome sequence data

Whole-genome sequence data was available for 168 of the most influential boars with many offspring for 10 commercial Dutch and Norwegian (Topigs Norsvin) lines, including 36 individuals originating from the Landrace breed, 39 from the Large White breed, 60 from the Duroc breed, 16 from a synthetic breed, 13 from the Pietrain breed, one Large White Dutch Landrace crossbred animal, and three animals of unknown origin. The reference population included 12 DL-line animals and 32 LW animals, corresponding to the target populations. One DL-line animal and nine LW-line animals had both whole-genome sequence data and high-density genotypes.

### Variant calling

Raw sequence data were mapped to the pig genome build Sscrofa10.2 (Ensembl72) [13] using the Burrows-Wheeler Aligner (BWA)-mem algorithm [14]. The average sequence coverage across the complete reference population was 11.6 fold. SNPs, short insertions and deletions were called with the GATK unified genotype-caller [15] for the complete reference population using default settings, but, in addition, the standard minimum confidence threshold was set to 30.0, the standard emittance confidence threshold was set to 20.0, and the target coverage threshold for down-sampling to coverage was set to 200. Subsequently, all detected variants were filtered using VCFtools [16], retaining variants with read depth values (per individual) ranging from 4 to 35 and variants with an overall Phred Quality score higher than 20, excluding variants with more than 20% missing data, and removing insertions and deletions. In addition, the variants were thinned such that the distance between variant sites was not shorter than 3 bp. The final step included phasing the reference population and imputing missing genotypes in the sequence data using Beagle 4.1 with 10 phasing iterations [17].

### Imputation to iWGS

Imputation of the 80 K genotypes of the DL-line and the 660 K genotypes of the LW-line to the iWGS was performed with Beagle 4.1 [17] using the multi-line reference population. The default parameter settings for Beagle were used, except for setting the effective population size to 300 instead of the default of 1 million [18] because it is much smaller in livestock than in humans [19, 20]. The accuracy of imputation at the SNP level was assessed by the Beagle R^2^, which is the estimated squared correlation of the genotype score with the true genotype [21].

For further analyses, the same quality controls as for the 80 K and 660 K genotypes were applied to the iWGS data but one additional quality filter was applied to the iWGS scores to account for possible imputation errors by removing SNPs with a Beagle R^2^ ≤ 0.6. This threshold was chosen to maintain a balance between the average imputation accuracy and the number of SNPs removed. This filter was not applied to allele dosage scores, which were coded as any real value between 0 and 2, because dosage scores account for imputation uncertainty.

### Genome-wide association study

A single SNP GWAS was performed with a mixed linear model using GCTA version 1.25.2 [22, 23]. The GWAS with iWGS dosage scores was performed with an adapted version of GCTA (patches provided and described in Additional file 1). The model was as follows:

$${\mathbf{y}} = {\mathbf{1}}\mu + {\mathbf{x}}b + {\mathbf{u}} + {\mathbf{e}},$$

where ${\mathbf{y}}$ is a vector of phenotypes, *b* is the fixed effect of the SNP tested for association, ${\mathbf{x}}$ is a vector containing the genotype scores or dosage scores, ${\mathbf{u}}$ is a vector of random polygenetic effects, and ${\mathbf{e}}$ is a vector of residuals, which were assumed followed a normal distribution $N\left( {{\mathbf{0}},{\mathbf{I}}\sigma_{e}^{2} } \right)$ with *σ*_*e*_^2^ as error variance. The vector ${\mathbf{u}}$ was assumed to follow a normal distribution $N\left( {{\mathbf{0}},{\mathbf{G}}\sigma_{g}^{2} } \right)$, where ${\mathbf{G}}$ is the genomic relationship matrix and *σ*_*g*_^2^ is the genetic variance. To account for population structure and to prevent possible bias from fitting the same SNP twice, the genomic relationship matrix was computed based on all SNPs except those that were on the same chromosome as the tested SNP [24]. The genomic relationship matrices were computed as follows [23]:

$${\mathbf{G}}_{ik} = \frac{1}{N}\mathop \sum \limits_{i} {\mathbf{G}}_{ijk} = \left\{ {\begin{array}{*{20}c} {\frac{1}{N}\mathop \sum \limits_{i} \frac{{(x_{ij} - 2p_{i} )(x_{ik} - 2p_{i} )}}{{2p_{i} \left( {1 - p_{i} } \right)}},{\text{j}} \ne {\text{k}}} \\ {1 + \frac{1}{N}\mathop \sum \limits_{i} \frac{{x_{ij}^{2} - \left( {1 + 2p_{i} } \right)x_{ij} + 2p_{i}^{2} }}{{2p_{i} \left( {1 - p_{i} } \right)}},{\text{j}} = {\text{k}}} \\ \end{array} } \right\},$$

where *x*_*ij*_(*x*_*ik*_) is the genotype coded 0, 1, or 2 for the *i*th SNP of the *j*(*k*)th individual, *N* is the total number of markers, and *p*_*i*_ is the MAF of the *i*th SN*P*. The percentage of phenotypic variance explained by the *i*th SNP was estimated as $\frac{{2p_{i} \left( {1 - p_{i} } \right)*\hat{b}^{2} }}{{var\left( {phenotype} \right)}}*100$, where $\hat{b}$ is the estimated allele substitution effect.

### Significance testing

To account for population structure, the GWAS p-values for each SNP density were adjusted by their corresponding genomic inflation factors [25], which were calculated for each SNP density as the median of the observed Chi squared test statistics divided by the expected median of the corresponding Chi squared distribution assuming 1 degree of freedom. The Chi square test statistics were calculated from the p-values. Significance thresholds were then established by applying a Bonferroni correction by dividing the expected probability of a type-1 error (α = 0.05) by the number of independent tests. Following Duggal et al. [26] and Ricard et al. [27], we assumed that the number of independent tests was equal to the number of independent chromosome segments, which was calculated using the formula proposed by Goddard et al. [22]. For the LW-line, the number of independent chromosomal segments was 776.4, 648.1 and 782.3 for the 80 K and 660 K SNP panels and the iWGS genotype scores, respectively. For the DL-line, the number of independent chromosomal segments was 249.5, 277.4, and 280.6 for the 80 K and 660 K SNP panels and the iWGS genotype scores, respectively. Since, within a line, the numbers of independent chromosomal segments were similar between SNP densities, the same significance threshold was used for all densities within a line. As a result, a SNP was considered significant when it was associated with a − log_10_ (*p* value) higher than 4.2 and 3.7 for the LW-line and the DL-line, respectively.

To identify QTL regions, SNPs on each chromosome were ranked based on their *p* values and, starting with the SNP with the highest − log_10_ (*p* value), all significant SNPs within a 0.5-Mb region to the left and right of the SNP were assigned to that QTL region. This procedure was repeated until all significant SNPs were assigned to a QTL region. We chose this definition for a QTL region and assumed that significant SNPs that are more than 0.5 Mb apart belong to independent QTL regions because the average LD of commercial pig lines decreases to less than 0.3 when the SNPs are more than 0.5 Mb apart [28–30].

## Results

### Genotypes

An overview of the number of SNPs available for each SNP density and line is in Table 1. In total, 26.1 × 10^6^ SNPs were available in the sequenced reference animals, of which 17.6 × 10^6^ and 21.7 × 10^6^ segregated in the iWGS data for the DL-line and the LW-line, respectively. After quality control, 5.4 × 10^6^ SNPs with iWGS dosage scores and 3.5 × 10^6^ SNPs with genotype scores remained for the LW-line, and 5.8 × 10^6^ SNPs with iWGS dosage scores and 2.5 × 10^6^ SNPs with genotype scores remained for the DL-line. Not all SNPs on the SNP panels were present in the WGS data, i.e., for the LW-line, 91.2% of the 80 K SNPs and 88.8% of the 660 K SNPs were present in the WGS data, and for the DL-line 89.7% of the 80 K SNPs were present in the WGS data.

**Table 1:** Number of SNPs used for GWAS after quality control for different SNP densities and use of imputed whole-genome sequence dosage or genotype scores

	DL-line	LW-line
80 K SNP genotypes	38,228	34,588
660 K SNP genotypes	311,888	491,169
iWGS_genotype score	2,495,861	3,476,936
iWGS_dosage score	5,841,784	5,453,881

### Imputation accuracy

**Fig. 1:**
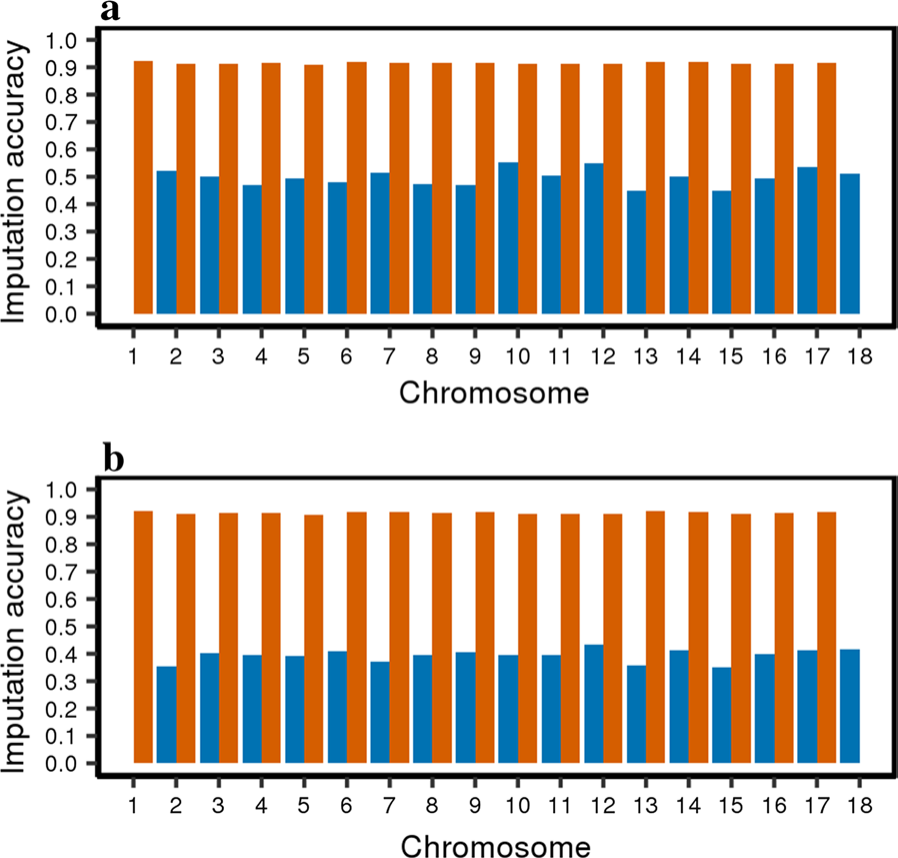
Accuracy of imputation to whole-genome sequence by chromosome for the LW-line (**a**) and the DL-line (**b**). Accuracy of imputation based on Beagle R^2^ before (blue) and after (orange) filtering

Before filtering, the average Beagle R^2^ (measure of imputation accuracy) across the whole genome was relatively low. The LW-line 660 K genotypes were imputed to iWGS with an average Beagle R^2^ of 0.49; after removing SNPs with an Beagle R^2^ lower than 0.6, the average R^2^ was equal to 0.93. The DL-line 80 K genotypes were imputed to iWGS with an average Beagle R^2^ of 0.39 and of 0.84 after removing SNPs with an imputation Beagle R^2^ < 0.6. Before filtering and quality control, the Beagle R^2^ varied between (Fig. 1) and within chromosomes (see Additional file 2: Figures S1 and S2). For the LW-line, the lowest and highest Beagle R^2^ were obtained for chromosome 13 (0.45) and 10 (0.55), respectively. For the DL-line, the lowest and highest R^2^ were obtained for chromosome 15 (0.35) and 12 (0.43), respectively. Beagle R^2^ also varied along each chromosome for both lines. Some regions had a low SNP coverage of SNPs, resulting in lower Beagle R^2^ in neighbouring regions, as illustrated by the region around 105.5 Mb on chromosome 7 for the DL-line (see Additional file 2: Figures S1 and S2).

**Fig. 2:**
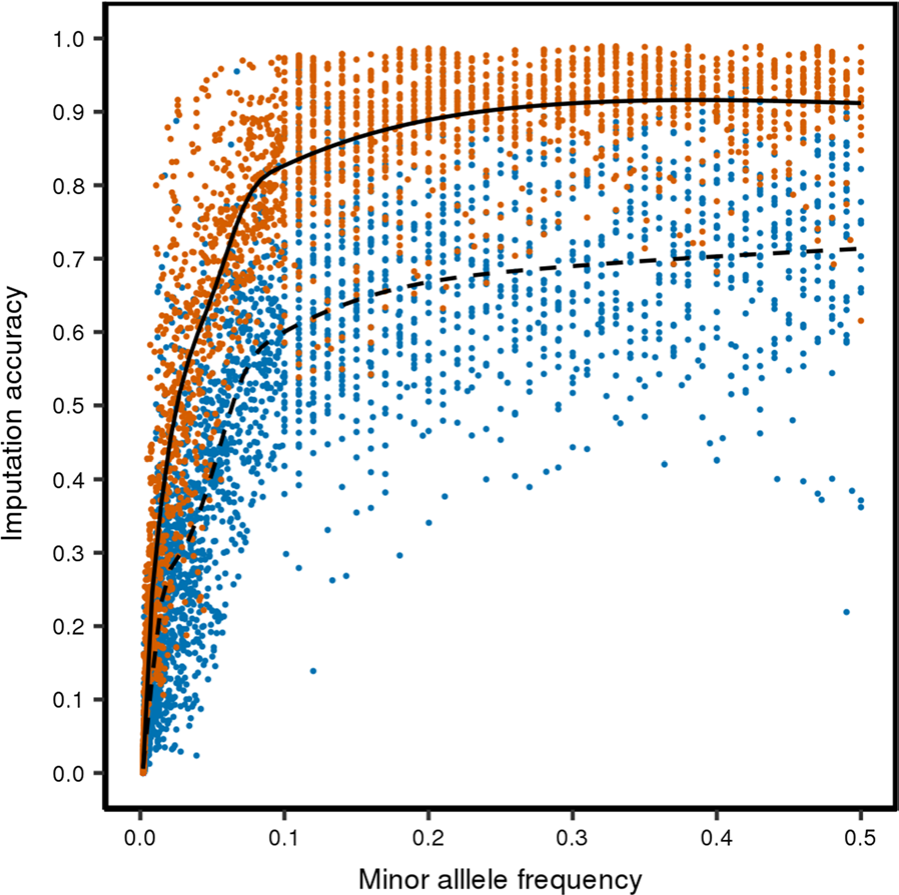
Accuracy of imputation to whole-genome sequence versus minor allele frequency. Imputation accuracy based on Beagle R^2^ is plotted against minor allele frequency of the SNPs on chromosome 7 for the LW-line (orange dots and solid line) and the DL-line (blue dots and dashed line). The SNPs were divided into bins of 500 SNPs and each dot represents one bin

Imputation accuracy, i.e. Beagle R^2^, increased with increasing MAF based on the iWGS genotype scores for each target line (Fig. 2). The most pronounced increase in accuracy of imputation was observed for the 0.00–0.15 MAF range. When MAF increased above 0.15, Beagle R^2^ reached a plateau at about 0.9 for the LW-line and 0.7 for the DL-line. After filtering on imputation accuracy, most SNPs with a very low MAF (< 0.01) were removed; the median MAF was 0.05 before filtering and 0.17 after filtering.

### GWAS

**Fig. 3:**
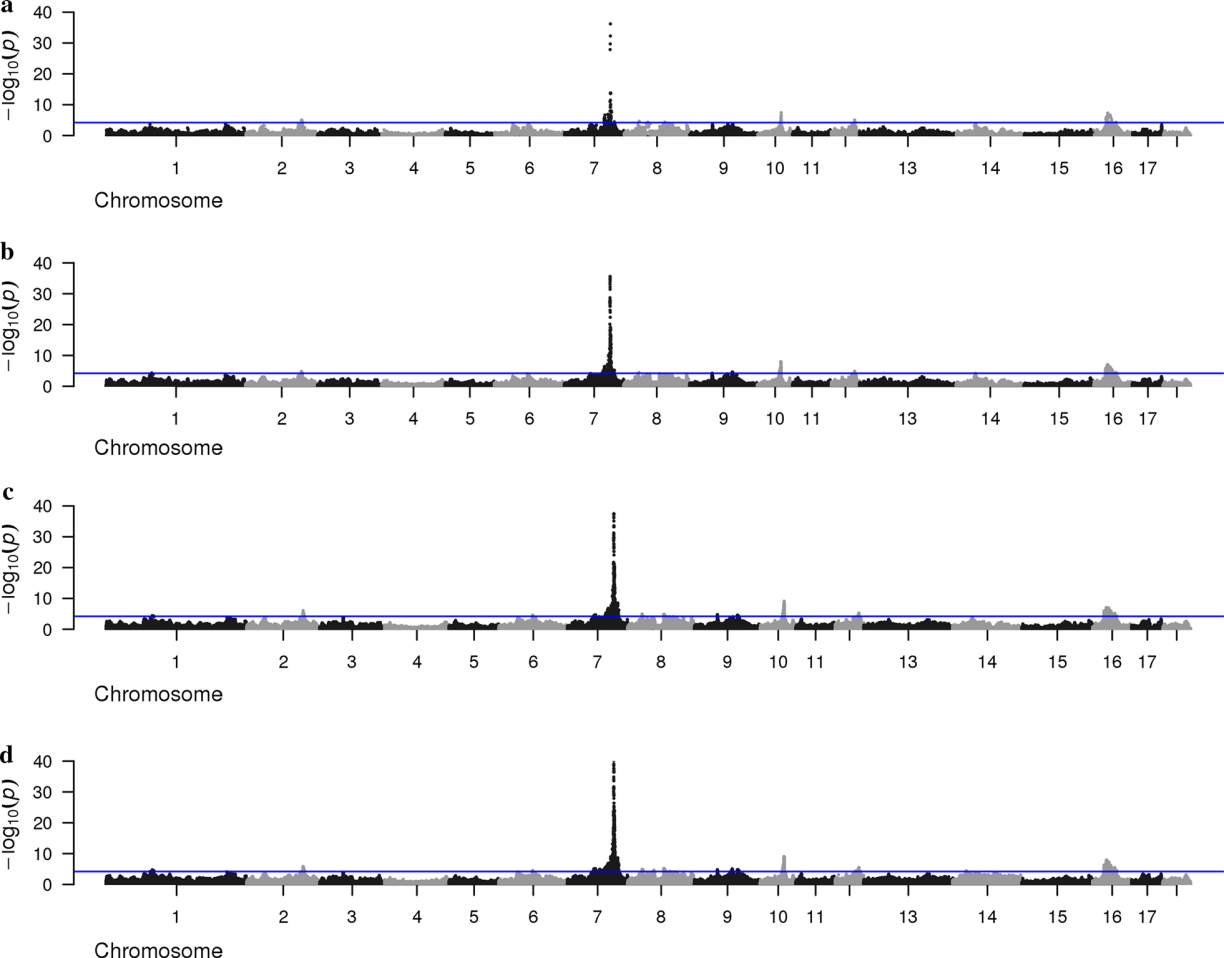
Manhattan plot for number of teats in the LW-line using different SNP densities. Manhattan plot for number teats in the LW-line population using **a** 80 K chip, **b** 660 K chip, **c** iWGS genotype scores and **d** iWGS with dosage scores

**Fig. 4:**
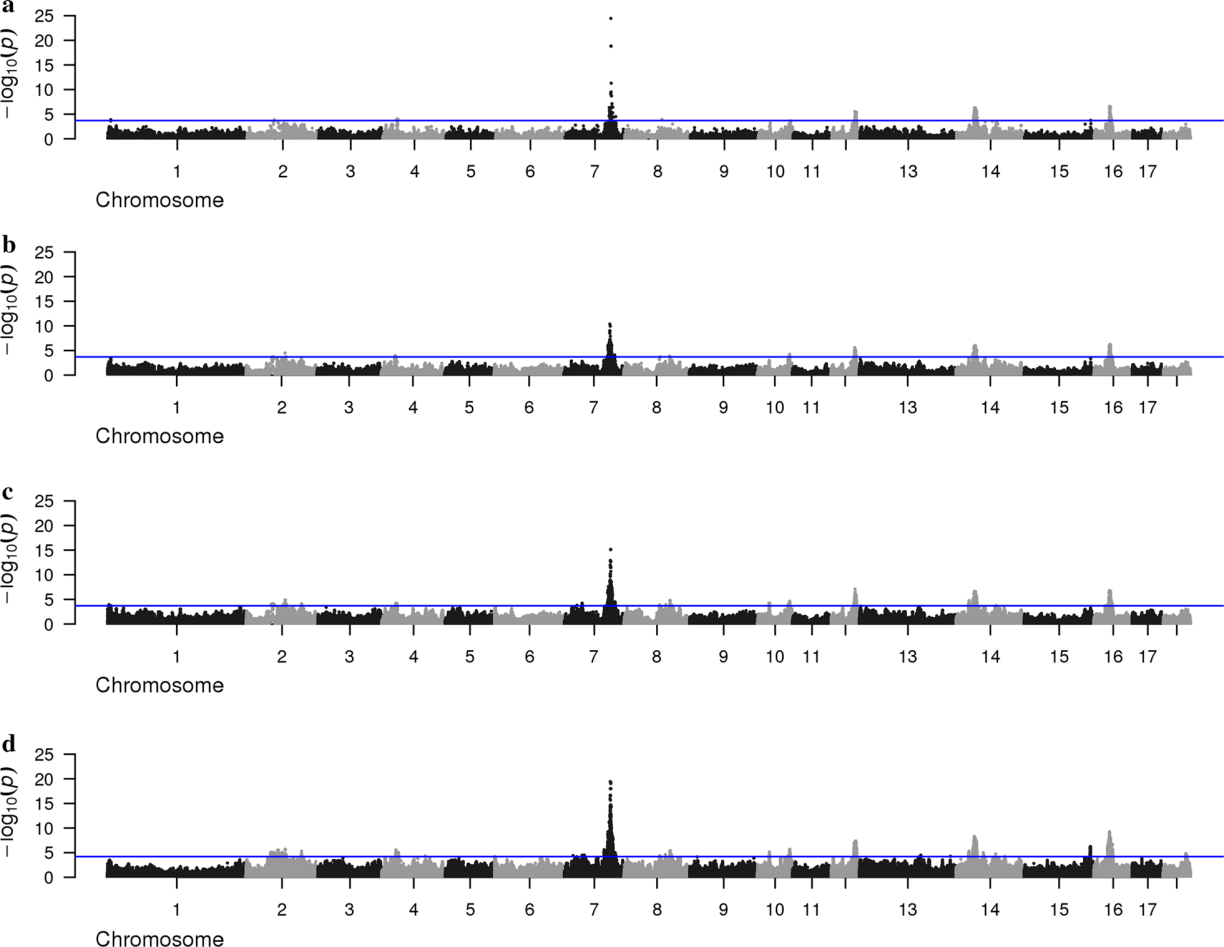
Manhattan plot for number of teats in the DL-line using different SNP densities. Manhattan plot for number teats of the DL-line population using **a** 80 K chip, **b** 660 K chip and **c** iWGS genotype scores, **d** iWGS dosage scores

An overview of the GWAS results based on the 80 K and 660 K SNP genotypes (based on iWGS for the DL-line), and based on the iWGS genotype and dosage scores is in Figs. 3 and 4 for the DL-line and LW-line, respectively. For both lines, the average number of QTL regions increased with increasing SNP density. For the LW-line, 37 and 104 QTL regions were identified with the 80 K SNP genotypes and the iWGS genotype scores, respectively. For the DL-line, the number of QTL regions detected increased from 48 with the 80 K SNP genotypes to 94 with iWGS genotype scores. Of the QTL detected based on iWGS genotype scores, 48.9 and 64.4% were not identified with the 80 K SNP genotypes for the DL-line and the LW-line, respectively. Even more QTL regions were identified when iWGS dosage scores instead of genotype scores were used (Table 2); for the LW-line and DL-line, the number of QTL regions detected increased to 132 and 217, respectively.

**Table 2:** Descriptive statistics of results of the GWAS for the two lines using different SNP densities and imputed whole-genome sequence dosage or genotype scores

	Threshold	Number of QTL regions	Genomic inflation factors
DL-line			
80 K SNP genotypes	3.7	48	1.80
660 K SNP genotypes	3.7	48	2.05
iWGS_genotype scores	3.7	94	1.81
iWGS_dosage scores	3.7	217	1.51
LW-line			
80 K	4.2	37	3.50
660 K	4.2	60	3.63
iWGS_genotype	4.2	104	3.45
iWGS_dosage	4.2	132	3.18

The Manhattan plots for each SNP density and for both lines showed a clear peak on chromosome 7 (Figs. 3, 4), reaching a -log10(adjusted p-value) of at least 10 for each SNP density for both lines. The peak was located between 103 and 105 Mb but, within this window, the position of the most significant SNP differed between SNP densities. In addition, for the LW-line, strong significant QTL regions were identified on chromosomes 10 and 16 for all densities, and on chromosomes 2 and 12 for the higher SNP density. For the DL-line, strong significant QTL regions were identified on chromosome 12 and 16 for all densities, and on chromosomes 2 and 10 for the higher SNP density.

**Fig. 5:**
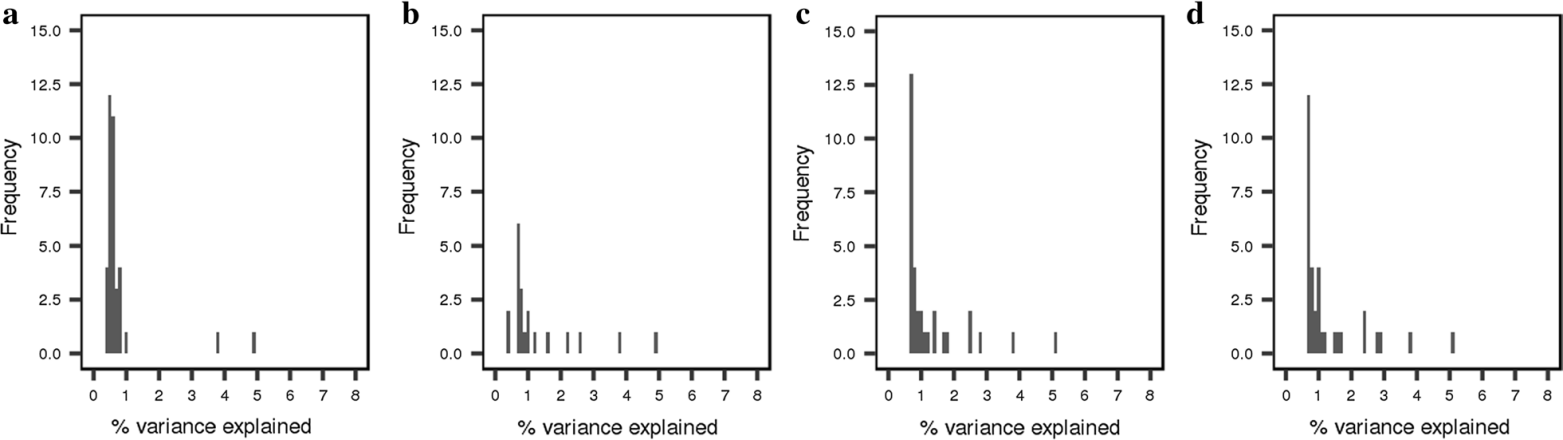
Distribution of the percentage of phenotypic variance explained by the QTL regions identified using different SNP densities for the LW-line. Distribution of the percentage of phenotypic variance explained by the QTL regions identified for the LW-line using **a** 80 K, **b** 660 K and **c** iWGS genotype scores, **d** iWGS dosage scores. The percentage of phenotypic variance explained was calculated as follows: $\frac{{2p_{i} \left( {1 - p_{i} } \right)*\hat{b}^{2} }}{{var\left( {phenotype} \right)}}*100$, where the phenotypic variance was 1.08

**Fig. 6:**
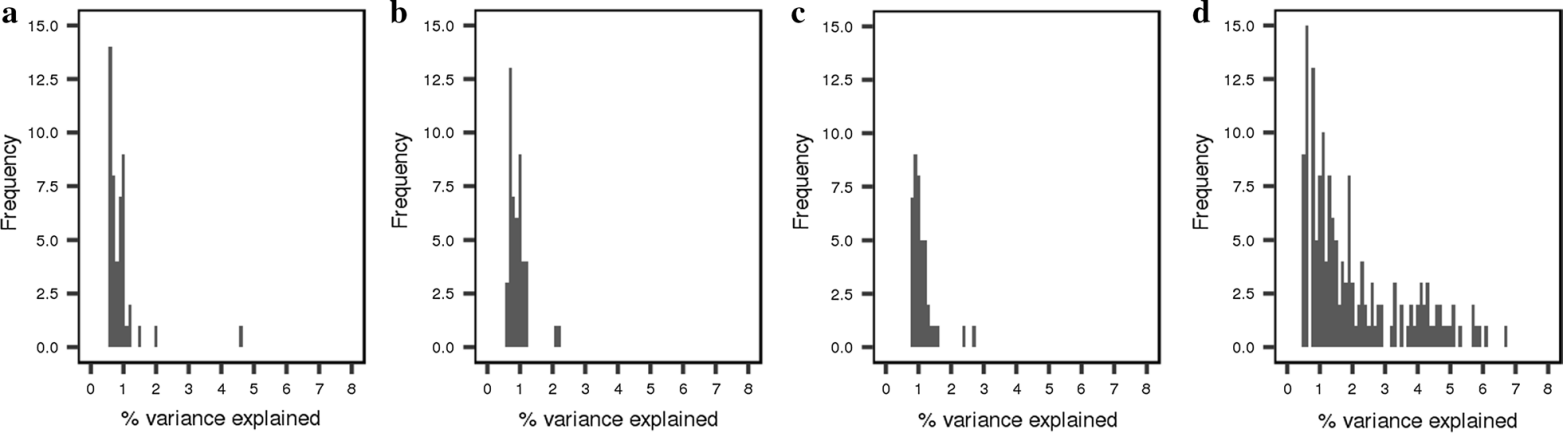
Distribution of the percentage of phenotypic variance explained by the QTL regions identified using different SNP densities for the DL-line. Distribution of the percentage of phenotypic variance explained by the QTL regions identified for the DL-line using **a** 80 K, **b** 660 K and **c** iWGS genotype scores, **d** iWGS dosage scores. The percentage of phenotypic variance explained was calculated as follows: $\frac{{2p_{i} \left( {1 - p_{i} } \right)*\hat{b}^{2} }}{{var\left( {phenotype} \right)}}*100$, where the phenotypic variance was 0.96

Along with the number of significant QTL regions increasing with increasing SNP density, the number of QTL regions that explained a higher percentage of the phenotypic variance increased (Figs. 5, 6). For example, for the DL-line, the number of QTL that explained more than 1% of the phenotypic variance increased from 22 to 123 for the iWGS dosage scores versus 80 K genotypes. However, it should be noted that the percentage of variance explained, as computed here, is not cumulative because SNPs were tested one at a time and, therefore, the estimated effects of neighbouring SNPs were not independent due to LD.

Very high genomic inflation factors were observed, especially for the LW-line (> 3). For both lines, genomic inflation factors slightly increased as SNP density increased from 80 K to 660 K and then dropped to the same level as for the 80 K chip when iWGS genotype scores were used (Table 2). With iWGS dosage scores, genomic inflation factors decreased even more, i.e., from 1.80 for 80 K SNP genotypes to 1.51 for iWGS dosage scores for the DL-line, and from 3.50 for 80 K to 3.18 for iWGS dosage scores for the LW-line. Thus, inflation of the test statistic was lowest when dosage scores were used.

## Discussion

The objective of this study was to investigate the accuracy of imputation to WGS in two pig lines when using a multi-line reference population and the numbers of sequenced animals that belonged to the target lines were small, and subsequently, to investigate the effect of using the resulting imputed WGS genotype or dosage scores in a GWAS. Imputation from 80 K or 660 K to WGS using a multi-line reference population resulted in only 40 to 50% of the SNPs having a Beagle R^2^ higher than 0.60 in the iWGS data. Nonetheless, when accounting for imputation inaccuracy by filtering iWGS genotypes or by using dosage scores, the number of QTL detected and the estimated proportion of phenotypic variance explained by these were larger compared to when conducting the GWAS using lower density SNP chip genotypes, especially when using iWGS dosage scores. In the following section, the factors that influenced imputation accuracy are discussed, followed by a discussion on the effect of using iWGS genotype or dosage scores on the results of GWAS.

### Factors affecting accuracy of imputation

The average Beagle R^2^ found in this study was 0.39 for the DL-line and 0.49 for the LW-line, which were relatively low compared to other studies that have used multi-line reference populations but with a larger size (more than 242 individuals) [5–7], but similar to the accuracy of 0.46 obtained with 90 sequenced Holstein bulls by van Binsbergen et al. [4]. In our study, 168 individuals were included in the reference population and not all haplotypes present in our target populations were represented in the reference population, which increased imputation errors [4, 7]. Van Binsbergen et al. [4] investigated imputation accuracy in three scenarios that differed in the number of animals in a single breed reference population. They showed that for imputation from 50 K to iWGS, the accuracy was 0.37 with a reference population of 45 Holstein cows, and that it increased to 0.46 when the reference population increased to 90 Holstein cows. A similar increase of imputation accuracy was observed when imputing from 777 K to iWGS. These imputation accuracies were comparable or even higher than those found in our study, although we used a larger reference population. However, our reference population consisted of animals from 10 lines and the two target populations were only represented by 12 DL-line animals and 32 LW-line animals. The latter is probably the main reason for the disappointing imputation accuracy obtained in this study.

Our hypothesis was that adding animals from other lines and using a multi-line reference population would improve imputation accuracy, which was demonstrated previously especially for SNPs with a low MAF in the target population but that are segregating in other breeds [4, 6, 31–33]. For example, Bouwman and Veerkamp [6] showed that adding 60 individuals from the Jersey, Brown Swiss and Nordic Red Dairy cattle breeds to a reference population of 20 Holstein individuals increased the imputation accuracy of 777 K genotypes to iWGS for Holsteins from 0.71 to 0.83. In our study, the original small reference population was augmented by adding animals from other lines but imputation accuracy was still low for many chromosome regions. The genetic distances of the target populations with the other lines in the reference population were maybe too large because of, for example, different breeding goals. Given the small number of reference animals from the target lines (12 DL-line and 32 LW-line), accuracy of imputation would probably benefit more from additional sequenced DL-line and LW-line animals than from additional animals of other lines.

The imputation accuracy was lower for the DL-line than for the LW-line. Apart from the fact that there were only 12 DL-line individuals in the reference population compared to 32 LW-line individuals, the difference between starting density (i.e. 80 K genotypes for the DL-line and 660 K (imputed) genotypes for the LW-line) and target density (i.e. WGS) also contributed to the lower accuracy for the DL-line versus the LW-line. With a lower density SNP panel, the LD between the SNPs on the genotyping panel and WGS is lower and there is less information to identify shared haplotypes precisely, which increases the uncertainty of imputed genotypes [4, 33]. Stepwise imputation from 80 to 660 K to iWGS, instead of from 80 K to iWGS directly, as suggested by van Binsbergen et al. [4], and as performed for the LW-line, could improve imputation accuracy for the DL-line. However, this was not possible here because the number of available 660 K genotypes for the DL-line was not sufficient.

Other possible reasons for the difference in imputation accuracies between the two lines are differences in population structure or genetic architecture between the lines. For example, selection or different effective population sizes could have resulted in different LD decays and numbers of independent chromosome segments between the lines [34]. Populations with a smaller number of independent chromosome segments are expected to have a higher imputation accuracy because they are expected to have less LD decay across the genome and a greater number of shared haplotypes. In this study, the DL-line had a smaller number of independent chromosomal segments (280.6) than the LW-line (782.3), which was expected to increase imputation accuracy for the DL-Line. However, due to other factors such as the starting density and smaller representation in the reference population, we did not observe a higher imputation accuracy for the DL-Line.

In addition to the above-mentioned factors, imputation accuracy could also be affected by the unequal distribution of SNPs on the genotyping array along the genome and by mapping errors. The latter complicate imputation because incorrect positions of SNPs lead to errors in haplotypes and LD structure in the region they are incorrectly mapped to. Thus, an improved reference genome should increase imputation accuracy [1, 35].

### GWAS with iWGS genotype and dosage scores

Iso-Touru et al. [36] and Daetwyler et al. [1] identified new QTL when using iWGS genotype and dosage scores compared to SNP panel genotypes. Similarly, we also found new QTL regions when iWGS genotype scores were used. For the DL-line and the LW-line, 48.9 and 64.4%, respectively, of the QTL detected with iWGS genotype scores were not identified with the 80 K SNP genotypes. The QTL that were identified with all genotype densities were also reported in other GWAS studies that used DL-line or LW-line SNP genotypes [37–41]. In all these studies, the most significant QTL region was located on chromosome 7 and can be linked to the *Vert[n]in* gene. This gene is important for vertebral development and is positively correlated with number of teats in pigs [37, 39, 41]. Several new QTL regions identified with iWGS genotype and dosage scores include candidate genes for number of teats. For example, at approximately 125 Mb on chromosome 2, a QTL region was identified for the LW-line based on iWGS that includes the *casein kinase 1 gamma 3* gene, which plays a regulatory role in the *Wnt* signalling pathway, which is essential for early mammary gland formation [42–44]. For the DL-line, we found a QTL region on chromosome 2 at about 76.5 Mb that was close to another *casein kinase 1 gamma* gene (*casein kinase 1 gamma 2*).

Compared to the DL-line, more QTL regions were identified for the LW-line when 660 K genotypes and genotype scores were used but fewer QTL regions were identified when 80 K genotypes or dosage scores were used for GWAS. This was unexpected because the DL-line has a smaller population size and more DL-line iWGS genotype scores were removed because of low imputation accuracy. Therefore, the power to detect associations was expected to be lower in the DL-line than in the LW-line [45]. The larger number of QTL regions identified for the DL-line with 80 K genotypes and dosage scores might be because it has a smaller number of independent chromosomal segments (280.6) compared to the LW-line (782.3). A smaller number of independent chromosomal segments is expected to increase imputation accuracy because of less LD decay across the genome. As a result, power to detect associations might have been higher in the DL-line when using the 80 K genotypes and dosage scores. For 660 K and genotype scores, the power was lower in the DL-line compared to the LW-line because many SNPs with low imputation accuracy were removed. However, another possibility is that the smaller number of independent chromosomal segments for the DL-line, nay have resulted in the identification of a larger number of false positive QTL.

Of the 94 QTL regions found for the DL-line with iWGS genotype scores, 24% overlapped with QTL regions identified for the LW-line with iWGS genotype scores. Although power was limited in each line and we did not expect to detect each QTL in each line, differences might also be caused by differences in genetic architecture between the lines, such as MAF and LD patterns [29]. Comparing the MAF of the most significant SNPs that were identified in the DL-line with the same SNPs in the LW-line (see Additional file 2: Figure S3), clearly showed that the QTL regions had different MAF between the two lines. For example, a SNP on chromosome 8 located at 104.1 Mb had a MAF of 0.005 and 0.36 in the DL-line and LW-line, respectively. Assuming that the SNP is associated with the phenotype in both lines, its low MAF reduces the power to detect it in the DL-line.

Although a large number of new QTL were found using iWGS genotype, whether they are indeed new associations or artefacts of the definition of the QTL region can be questioned. Here, a QTL region was defined as the 0.5-Mb region to the left and right of the most significant SNP in a region because multiple studies have found that the average LD in commercial pig lines decreases below 0.3 when the SNPs are more than 0.5 Mb apart [28–30]. This definition may increase the number of QTL regions for iWGS because iWGS consists of many SNPs that are in very high LD. In addition, LD decay can vary across and within chromosomes [29] and, therefore, this definition can be too strict for some QTL regions and neighbouring regions could be merged into one region or the other way around. To test this, we increased the size of the QTL region to 1 Mb to the right and left of the most significant SNP in that region. This resulted in a reduction in the number of QTL detected with iWGS genotype scores from 104 to 64 for the DL-line and from 94 to 56 for the LW-line. Although this is a significant reduction, it is still larger than the number of QTL regions based on the original QTL region definition found with 80 K and 660 K.

In addition to the definition of the QTL region, the newly identified QTL regions could also be an artefact due to the significance threshold that we applied in this study. Here, the significance threshold was set by applying a Bonferroni correction using the number of independent chromosomal segments instead of the commonly used total number of SNPs, which does not take LD between SNPs into account. The number of independent chromosomal segments has often been used for Bonferroni correction for GWAS in human [26, 46, 47], plant [48–50], and animal [27, 51] genetics. However, so far there has been little consensus about the most appropriate approach for testing significance for GWAS. This should be a topic for future research.

Finally, even with low imputation accuracy, GWAS using iWGS can be beneficial in several cases. For example, the QTL regions identified in this study could help to pre-select SNPs to improve the accuracy of genomic predictions, as shown by [52–54]. In addition, QTL regions identified could be used as indicators for fine-mapping of possible interesting regions, even if they are based on poorly imputed WGS data. For example, SNPs that are in the detected QTL regions could be added to high-density SNP chips, or animals with extreme phenotypes could be sequenced for the detected QTL regions.

### Dosage scores

**Fig. 7:**
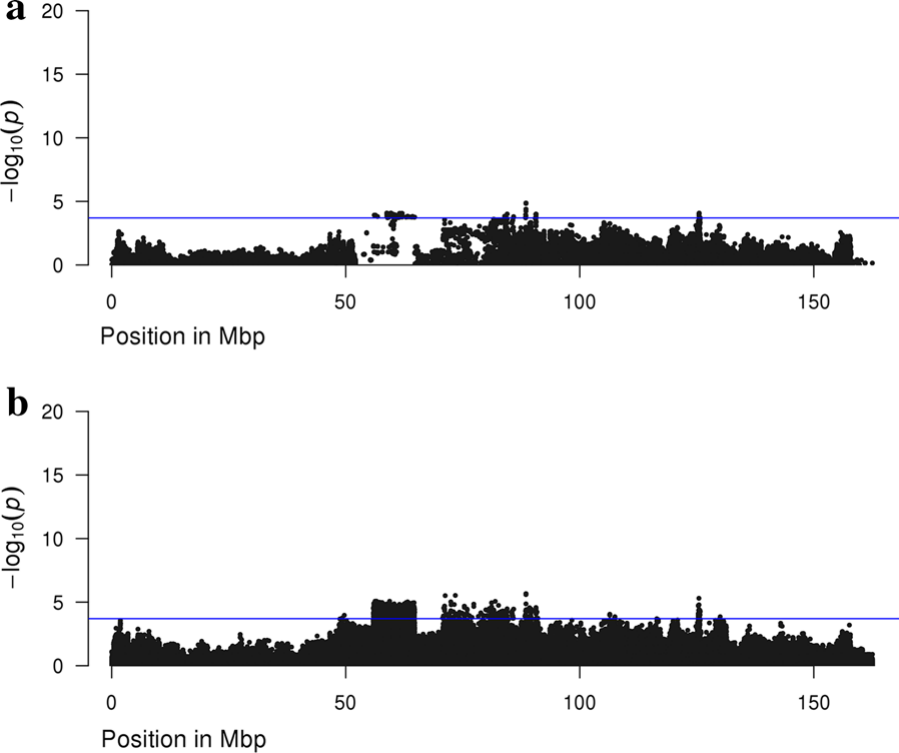
Manhattan plot for number of teats for the DL-line using imputed whole-genome sequence dosage or genotype scores, zoomed-in on chromosome 2. Manhattan plot for number teats of the DL-line population using **a** iWGS genotype scores, **b** iWGS dosage scores zoomed-in on chromosome 2

**Fig. 8:**
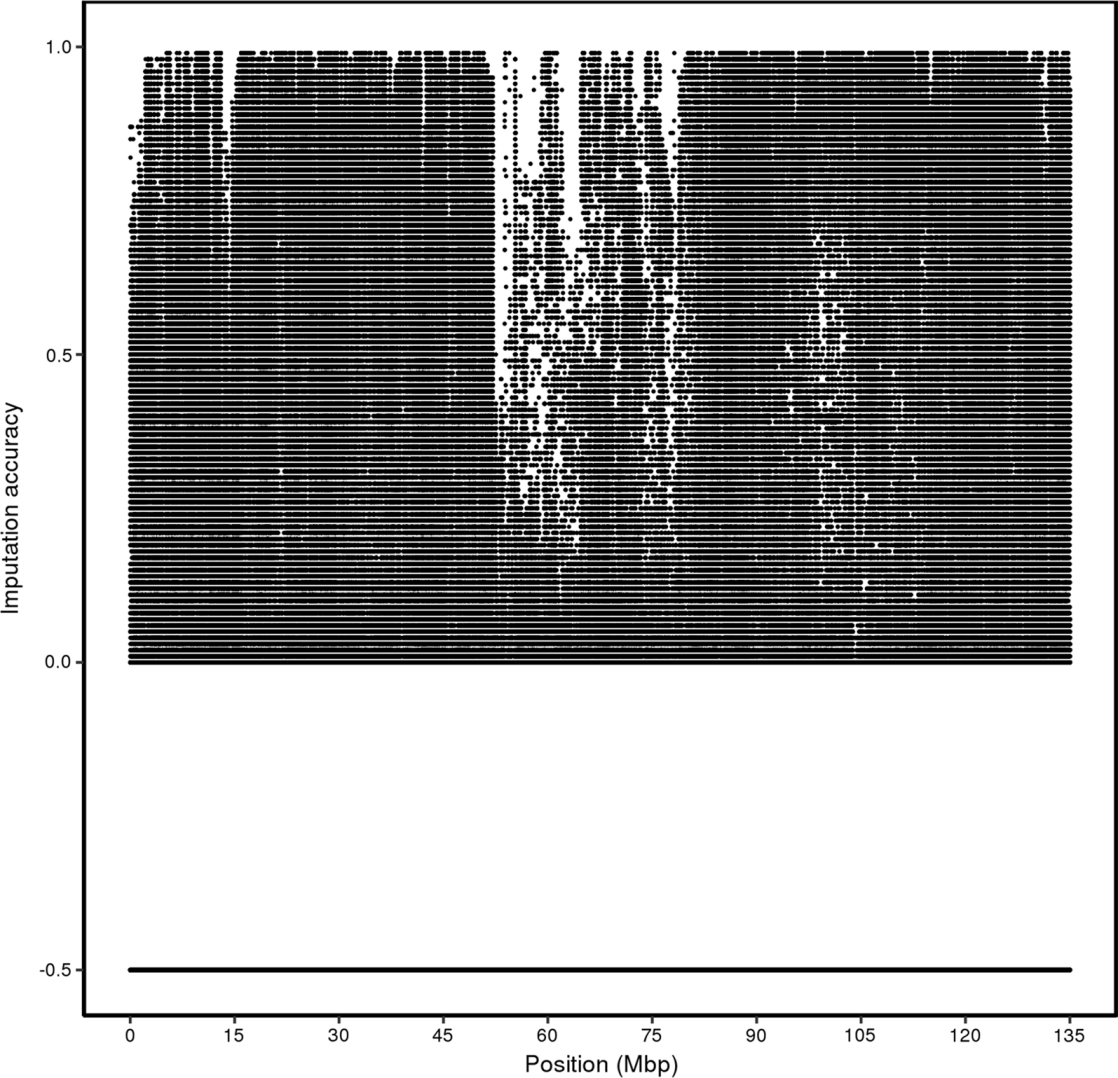
Distribution of the accuracy of imputation to whole-genome sequence along chromosome 2 for the DL-line

In this study, imputation accuracy by SNP, measured with Beagle R^2^, was relatively low and, therefore, many SNPs were inaccurately imputed. When using iWGS genotype scores, quality control measures removed around 50% of the SNPs from the analysis and we assumed that the remaining SNPs were called without imputation error. Using iWGS dosage scores in the GWAS model is another way of analysing poorly imputed data. Dosage scores are a posterior probability of having one of the three genotypes and, thus it accounts for the uncertainty of imputation to iWGS. In this study, we identified 56.7 and 26.9% more QTL regions for the DL-line and the LW-line, respectively, when dosage instead of genotype scores were used. Moreover, the most significant SNPs in the QTL regions explained more of the phenotypic variance when using dosage scores. Genotype scores may not capture as much phenotypic variation because some information is lost due to inaccurate imputation. For example, for the DL-line, a clear peak was detected on chromosome 2 at approximately 56 Mb with dosage scores but not with genotype scores (Fig. 7). In this region, many SNPs did not pass the quality control for the iWGS genotype score scenario, because imputation accuracy for this region was low (Fig. 8). In addition, the Manhattan plot based on iWGS genotype scores (Fig. 7) showed a very odd pattern, with an 8-Mb region from 56 to 64 Mb including SNPs that had the same significance level. This region displays high LD (i.e., with an average (± SD) LD r^2^ of 0.46 (± 0.07)), and thus the SNPs in this regions had approximately the same imputation accuracy and dosage scores, and thus, the same significance level.

### Genomic inflation factor

In this study, the genomic inflation factors were lower when using dosage scores compared to using 80 K and 660 K genotypes and iWGS genotype scores. The scenario using dosage scores included SNPs with a low imputation accuracy, and hence SNPs with a low MAF. In the other scenarios, there is ascertainment bias, which is caused by the preference of SNPs on a chip that are more likely to be common [55]. In addition, SNPs selected for iWGS genotype scores have ascertainment bias, because accurately imputed SNPs in general have a higher MAF. By (indirectly) selecting SNPs with a high MAF, it is easier to detect effects for these SNPs, and their surrounding SNPs, and therefore it is likely that more significant SNPs are found than expected based on the theoretical distribution of the test statistic distribution under the null hypothesis. Linkage disequilibrium between the SNPs will also result in more significant SNPs within the region that surrounds a causal variant. Both the higher frequency of SNPs with high MAF and long-range LD increase genomic inflation factors [56–58] and the rate of false positives. Use of dosage scores leads to less inflation of the test statistic because the ascertainment bias is partly removed, leading to less biased SNP effects compared to the use of lower density SNP chips and iWGS genotype scores.

## Conclusions

Use of a multi-line reference population resulted in relatively poor imputation accuracy to iWGS, with 52.7% of the SNPs on the 660 K array in the LW-line and 39.1% of the SNPs on the 80 K array in the DL-line having Beagle R^2^ lower than 0.6. Imputation from 660 K to iWGS was more accurate than imputation from 80 K to iWGS, which suggests that step-wise imputation, i.e. first imputing from 80 to 660 K and then from 660 K to iWGS, could increase the accuracy of imputation. Although imputation accuracy was poor, using iWGS instead of genotypes from a lower density SNP chip increased the number of detected QTL regions and the expected proportion of phenotypic variance that they explained. When using iWGS dosage scores instead of genotype scores, even more QTL regions were detected because all SNPs were used in the analysis and the uncertainty of imputation was taken into account by the model.

## Additional files

**Additional file 1.** The adapted subroutines in GCTA. Patches for the program GCTA (Version 1.26.0) to be able to run GWAS with dosage scores.

**Additional file 2.** Figures S1 and S2 contain the Beagle R^2^ of imputation on chromosome 7 for the LW-line and DL-line, respectively, and Figure S3 shows the minor allele frequency of the most significant SNPs identified in the DL-line with iWGS genotype scores plotted against their minor allele frequency in the LW-line.
