# Supplementary material for: Imputation to whole-genome sequence using multiple pig populations and its use in genome-wide association studies
Source: Genet Sel Evol. 2019 Jan 24;51:2. doi: 10.1186/s12711-019-0445-y (PMC6346588; doi:10.1186/s12711-019-0445-y)
Supplement: Supplementary file 2 — Additional file 2. Figures S1 and S2 contain the Beagle R2 of imputation on chromosome 7 for the LW-line and DL-line, respectively, and Figure S3 shows the minor allele frequency of the most significant SNPs identified in the DL-line with iWGS genotype scores plotted against their minor allele frequency in the LW-line. [file 12711_2019_445_MOESM2_ESM.docx]

**Supplementary material 2**

a.

**
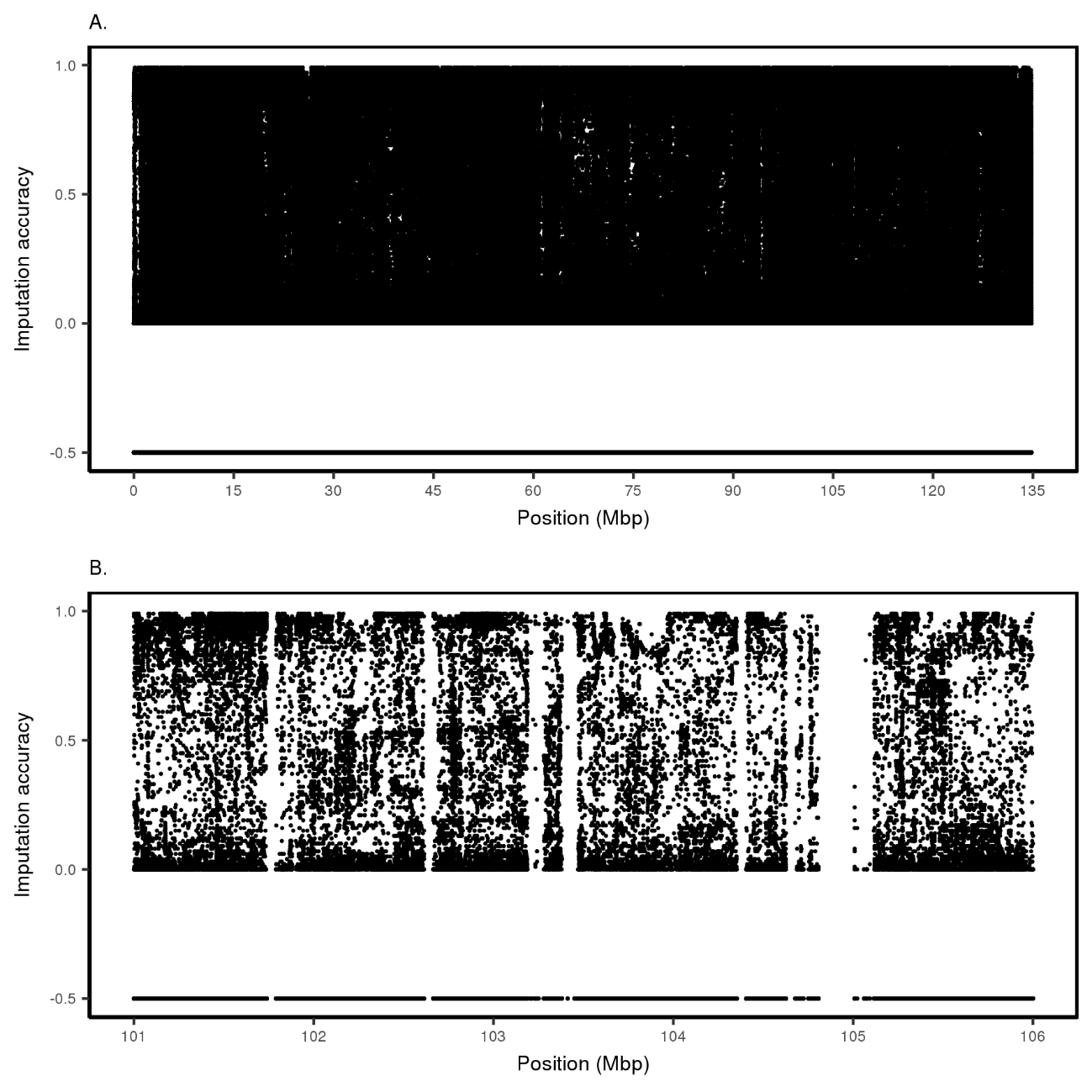
**

b.

**Figure S1. Beagle R^2^ of imputation on chromosome 7 for the LW-line**

The distribution of the Beagle R2 versus the location on chromosome 7 **(a)** for the whole chromosome and **(b)** for region between 101 and 106 MB on chromosome 7. The line at -0.5 indicates the position of the 80K SNPs on the chromosome.

**
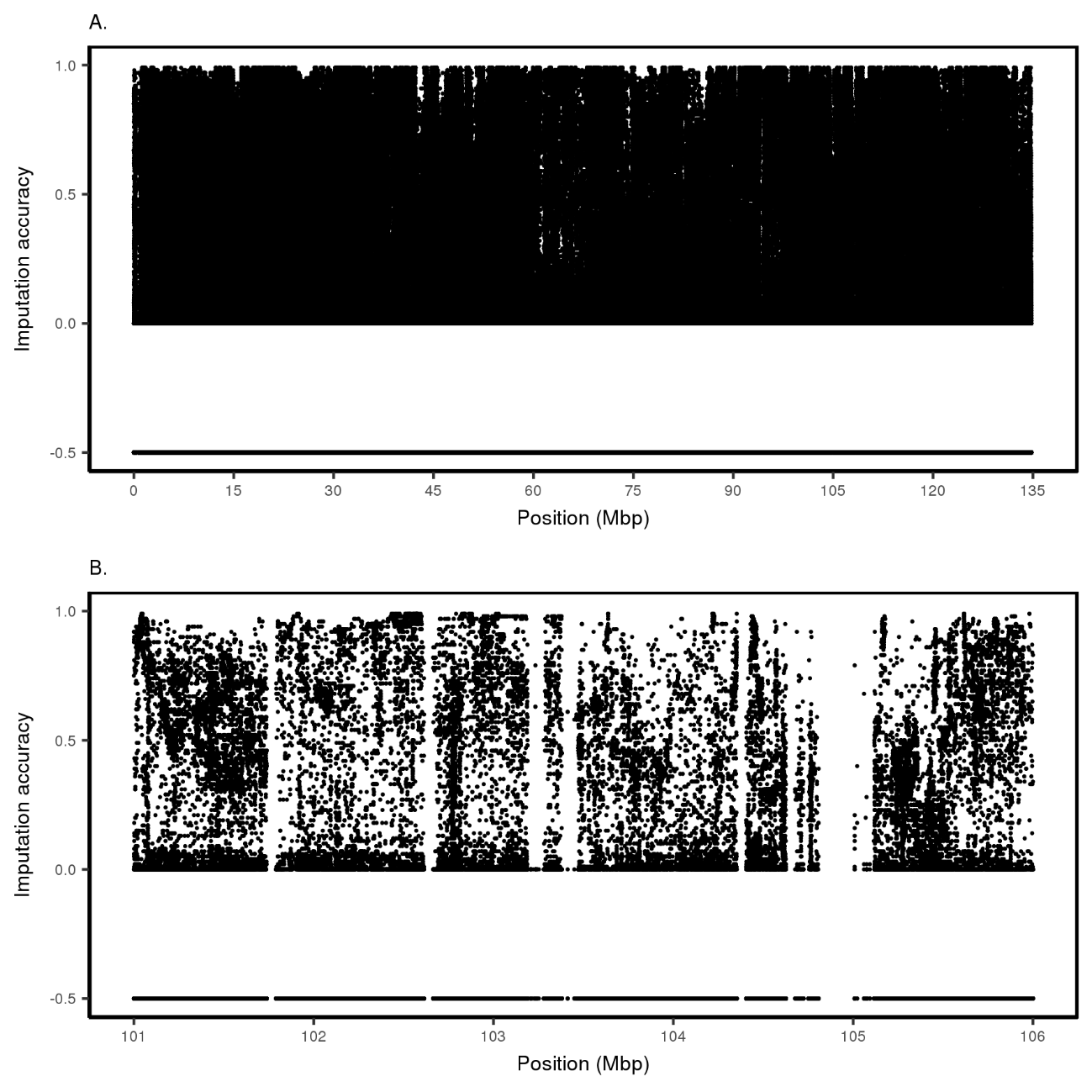
Figure S2. Beagle R^2^ of imputation on chromosome 7 for the DL-line**

b.

a.

The distribution of the Beagle R2 versus the location on chromosome 7 **(a)** for the whole chromosome and **(b)** for region between 101 and 105.5 MB on chromosome 7. The line at -0.5 indicates the position of the 660K SNPs on the chromosome.

**
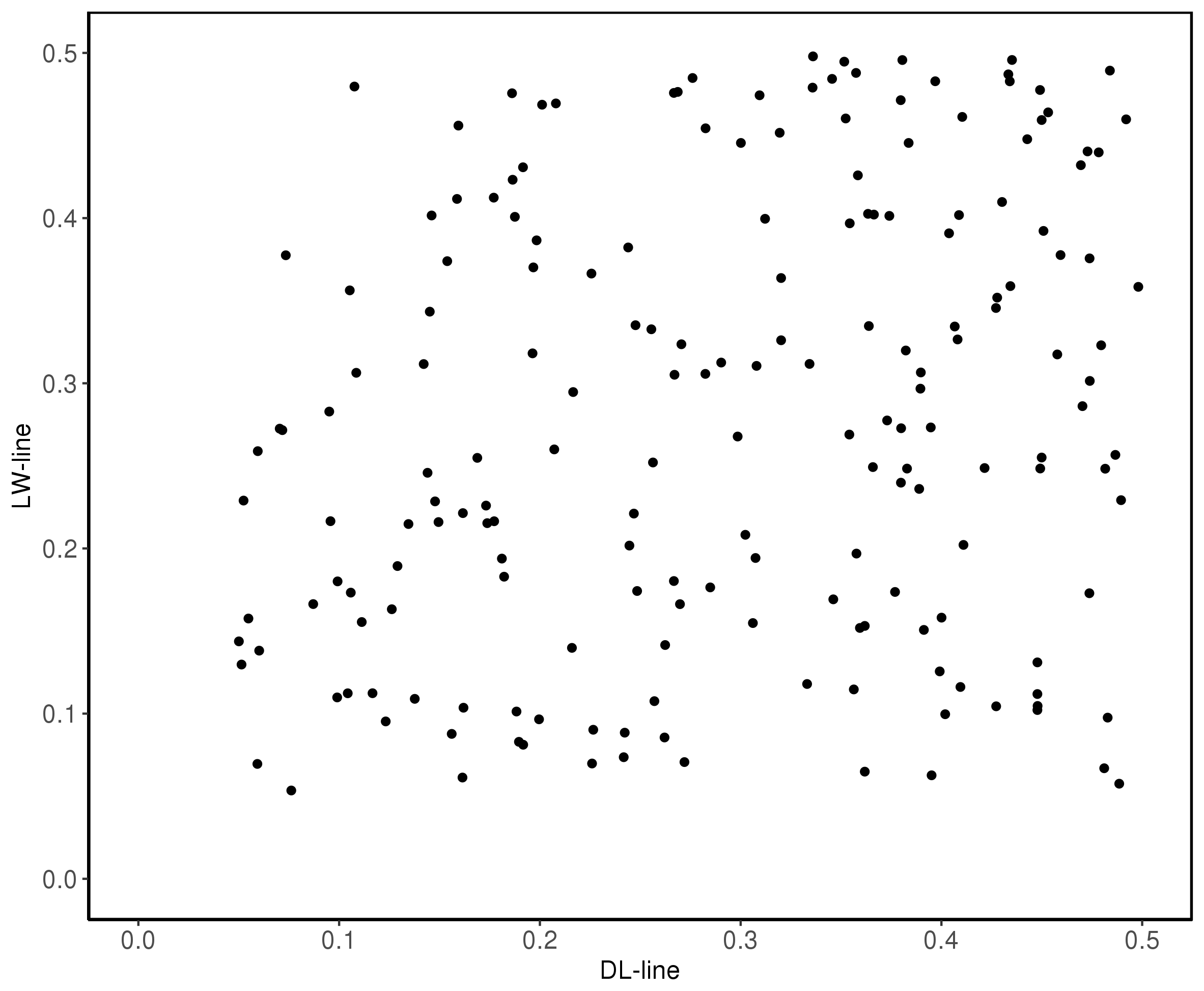
Figure S3. Minor allele frequency of the most significant SNP identified in DL-line with iWGS genotype scores plotted against their minor allele frequency in the LW-line**
